# Analysis of the microbiota of pregnant women in relation to weight gain during pregnancy – a pilot study

**DOI:** 10.3389/fcimb.2025.1655581

**Published:** 2025-08-29

**Authors:** Katarzyna Kosinska-Kaczynska, Magdalena Zgliczynska, Dominika Krawczyk, Magdalena Piatkowska, Aneta Balabas, Paweł Czarnowski, Krzysztof Goryca, Piotr Glinicki, Jerzy Ostrowski, Natalia Zeber-Lubecka

**Affiliations:** ^1^ Department of Obstetrics, Perinatology and Neonatology, Centre of Postgraduate Medical Education, Warsaw, Poland; ^2^ Department of Genetics, Maria Sklodowska-Curie National Research Institute of Oncology, Warsaw, Poland; ^3^ Department of Gastroenterology, Hepatology and Clinical Oncology, Centre of Postgraduate Medical Education, Warsaw, Poland; ^4^ Department of Endocrinology, Centre of Postgraduate Medical Education, Warsaw, Poland; ^5^ EndoLab Laboratory, Centre of Postgraduate Medical Education, Warsaw, Poland

**Keywords:** microbiota, pregnancy, gestational weight gain, microbiome, excessive gestational weight gain

## Abstract

**Introduction:**

Excessive body weight was associated with changes in individual microbiota. However, limited research on the impact of excessive gestational weight gain (GWG) revealed that microbiota patterns related to GWG differed from those linked to pregestational overweight or obesity.

**Aim:**

The aim was to compare differences in the microbiota of women in the third trimester of gestation who had excessive and non-excessive weight gain during pregnancy.

**Material and methods:**

Women with a singleton gestation at 34 + 0 weeks and normal pregestational body mass index were recruited to the study. Patients who were diagnosed with excessive weight gain formed the study group (n=11), while those with non-excessive weight gain formed the control group (n=10).

**Results:**

In cervico-vaginal samples, bacterial 16S rRNA gene sequencing demonstrated a decrease in alpha diversity, measured with the Shannon index, in the study group compared to the control group. While the difference was not statistically significant after correction for multiple testing, the Chao index showed a persistent trend toward reduced species richness in the study group. In stool samples, we identified 29 genera with differential representation between the groups, including nine overrepresented and ten underrepresented genera. The cervico-vaginal microbiota analysis detected 12 species distinguishing the study group from the controls, with four genera (*Ralstonia, Pandoraea, Kocuria*, and *Rhodobacteraceae unclassified*) being more prevalent in the study group. However, in both sites none difference was found to be statistically significant after p-value correction.

**Conclusions:**

Despite small sample size, we demonstrated slight trends in microbiota composition between groups. These suggest potential differences in microbial diversity and composition associated with excessive GWG, which supports further investigation.

## Introduction

The human microbiota consists of over 38 trillion cells, and the ratio of bacterial to human cells is estimated at 1.3:1, with most of them inhabiting the gut (Sender et al., 2016). It is believed that the microbiome has a significant impact on human health due to its multidirectional influence on metabolism, the immune and hormonal systems (Young, 2017). Therefore, the microbiome has the ability to directly and indirectly affect health, and dysbiosis accompanies almost every disease (Carding et al., 2015).

In the first trimester of pregnancy, the gut microbiome resembles that of the prepregnancy period, while in the following two trimesters it undergoes notable changes (Koren et al., 2012). We may observe an increased number of bacteria of the phyla Actinobacteria and Proteobacteria, as well as an increase in Verrucomicrobiota *(Akkermansia)*, Bifidobacterium, and Firmicutes, which were associated with energy storage (Koren et al., 2012; Gorczyca et al., 2022). Similarly to the gut, the microbiome of the vagina and cervix also changes during pregnancy. A significant reduction is mostly observed in the diversity of bacterial species in favor of an increase in the number of the *Lactobacillus* genus (DiGiulio et al., 2015; Serrano et al., 2019). The oral microflora remains relatively stable across pregnancy (Jang et al., 2021).

In recent decades, the problem of excess body weight has reached a pandemic level (Boutari and Mantzoros, 2022). According to Statistics Poland data, the percentage of obese women aged 30–39 accounted for 7.1% in 2009, 8.6% in 2014, and 9.8% in 2019, so an obvious upward trend could be noticed (Poland, 2019). The gut microbiome has a direct impact on carbohydrate and fat metabolism by inducing insulin resistance and regulating bile acid production and function (Gerard and Vidal, 2019). Short-chain fatty acids (SCFAs), produced by intestinal bacteria, affect the integrity of the intestinal barrier, decreasing inflammation, reducing insulin resistance and inducing the secretion of peptide YY hormone, which regulates the feeling of satiety (Holzer et al., 2012; Portincasa et al., 2022). Seemingly, a relationship may occur between overweight and obesity, and the composition of the microbiome. Available studies revealed a higher ratio of Firmicutes to Bacteroidetes accompanying excess body weight (Kasai et al., 2015; John and Mullin, 2016). A similar finding was reported in a group of pregnant women with excess prepregnancy body weight (Zacarias et al., 2018).

To date, little research has focused on the impact of excessive gestational weight gain (GWG). Stanislawski et al., who analyzed gut microbiota samples from 169 women shortly after delivery and from their infants up to two years of age, demonstrated that the bacterial taxa associated with excessive gestational weight gain (GWG) differ from those linked to pregestational overweight or obesity, and that these GWG-related associations are fewer in number and less consistent (Stanislawski et al., 2017).

The aim of the study was to compare the differences in the microbiota of the oral cavity, vagina, and stool, and stool metabolome between women in the third trimester of gestation who had excessive and proper weight gain during pregnancy.

## Materials and methods

Women with singleton gestation hospitalized at the Department of Obstetrics, Perinatology and Neonatology at the Centre of Postgraduate Medical Education were recruited to the study. The inclusion criteria were: maternal age of 18 years or older, single pregnancy, gestational age 34 + 0 weeks and beyond, a viable fetus, normal pregestational body mass index (BMI), informed consent given by the women. The exclusion criteria included: lack of informed consent to participate, history of intestinal surgery involving the use of an intestinal stoma or bariatric surgery, immunosuppression, human immunodeficiency virus infection or other conditions causing immune system dysfunction, intestinal dysbiosis syndrome, infectious diarrhea in the last 3 months before enrollment in the study, the use of probiotics, antibiotics or vaginal chemotherapeutics in the last 3 months before enrollment in the study, any vaginal medications in the last 3 months, severe chronic diseases: renal failure, heart failure, liver failure, pregestational diabetes or nonspecific bowel disease.

BMI was calculated as the ratio of body weight in kilograms to the square of body height in meters [kg/m (Young, 2017)]. The normal prepregnancy BMI was assumed to be 18.5–24.9 kg/m (Young, 2017) according to the values ​​recommended by the World Health Organization [WHO, (2000)]. The prepregnancy body weight was assessed to be the value declared by the study participant or the value entered in the first trimester of pregnancy medical records. The GWG was calculated as the difference between the body weight after completing 34 weeks of pregnancy and the prepregnancy weight. The proper GWG for women with a normal initial BMI was defined as 11.5–16 kg considering the recommendations of the Institute of Medicine and the National Research Council (Rasmussen and Yaktine, 2009). Excessive GWG was defined as a weight gain during pregnancy of over 16 kg. The study group was further divided into two subgroups of proper and excessive GWG.

The following anthropometric parameters of the study participants were collected: height, declared body weight before pregnancy, body weight at the time of enrollment. Samples of stool, secretions from the cervico-vaginal area and the oral vestibule were collected from each participant. All samples were collected during the subsequent 3 days after enrollment in the study.

### 16S rRNA gene sequencing and bioinformatic analysis

Cervico-vaginal and oral vestibule fluid samples were collected using 4N6FLOQSwabs™ (Thermo Fisher Scientific, USA). Stool samples were collected by the participants into sterile containers after detailed instruction on the collection technique. They were frozen at -20°C.

Bacterial genomic DNA was isolated using commercial kits for swabs (QIAamp DNA Mini Kit, Qiagen, Germany) and stool (QIAamp DNA Stool Mini Kit, Qiagen, Germany). Intestinal bacterial metabolites were extracted and analyzed using mass spectrometry combined with gas chromatography. The composition of the vaginal and cervical microbiome was assessed based on the sequencing of hypervariable fragments of the 16S rRNA gene using Ion Torrent technology (Thermo Fisher Scientific, USA). The bacterial 16S rRNA gene libraries were prepared using the Ion 16S™ Metagenomics Kit and the Ion Plus Fragment Library Kit (Thermo Fisher Scientific, USA). Sequencing targeted multiple hypervariable regions (V2-4–8 and V3-6,7–9) and was performed using the PGM™ Hi-Q™ View Sequencing Kit (Thermo Fisher Scientific, USA) reagents, following the manufacturer’s protocol, as described previously (Zeber-Lubecka et al., 2022; Kosinska-Kaczynska et al., 2025)

Unassigned BAM files were converted to the FASTQ format using the SamToFastq tool from the Picard suite. Subsequent analysis steps were performed using Mothur version 1.43 (Schloss et al., 2009). FASTQ files were converted to the FASTA format, retaining only sequences between 200–300 base pairs in length, with a minimum average quality score of 20 in a 50-base sliding window, and a maximum homopolymer length of 10 bases. Chimeric sequences were identified using the search algorithm with default parameters, referencing an internal sequence collection as the database (Rognes et al., 2016). Detected chimeras were removed, and the remaining 16S rRNA sequences were classified using the Wang method and the SILVA 16S rRNA bacterial reference database, with a threshold bootstrap set at 80% (Silva et al., 2006). To ensure comparability of diversity metrics across samples, rarefaction to a common sequencing depth was performed prior to alpha and beta diversity analyses. Alpha diversity analysis was conducted using the Shannon and Chao indices. Principal Coordinates Analysis (PCoA) was used to explore beta diversity and visualize differences in microbial community composition between the study and control groups. To assess the significance of clustering patterns, the Analysis of Similarities (ANOSIM) test was applied. Taxonomic abundance differences were evaluated using LinDA with default settings (Zhou et al., 2022). For differential abundance testing, taxonomic profiles were transformed to relative abundances and analyzed using LinDA with default settings, which include appropriate normalization procedures. The Mann-Whitney U test was used to evaluate diversity index differences between control and study group samples, while the Wilcoxon signed-rank test identified statistically significant differences in paired patient samples. Adjusted p-values (p adj) <0.05, controlling for the false discovery rate (FDR), were considered statistically significant.

### Analysis of bacterial metabolite concentrations

In addition to microbial community profiling, concentrations of bacterial metabolites, including short-chain fatty acids and amino acids (AAs), were measured using gas chromatography–mass spectrometry (GC-MS). For the analysis, 100 mg of fecal material was placed in a 2 mL tube containing ceramic beads specifically designed for environmental sample analysis (Ohaus Corporation, Parsippany, NJ, USA) and mechanically homogenized. The study employed commercial calibration standards for SCFAs, including formic acid, acetic acid, propanoic acid, butyric acid, isobutyric acid, pentanoic acid, isocaproic acid, and hexanoic acid, as well as for AAs (alanine, glycine, valine, leucine, isoleucine, proline, methionine, phenylalanine, and tyrosine) (Sigma-Aldrich, USA). Sample and standard derivatization was conducted with isobutyl chloroformate. The analysis was carried out using the Agilent 7000D Triple Quadrupole mass spectrometer, coupled with the GC 7890 system and G4513A autosampler (Agilent Technologies, Santa Clara, CA, USA), equipped with a VF-5ms column (30 m, 0.25 mm, 0.50 μm). Spectrometric data were collected in the full-scan mode from m/z 15 to 650 at 4.9 scans per second and processed using MassHunter software (Agilent Technologies, Santa Clara, CA, USA). The results were further statistically analyzed using GraphPad Prism biostatistical software.

The study protocol was approved by the local ethics committee at the Centre of Postgraduate Medical Education (number 46/2022).

Statistical analysis of study participant characteristics as well as the comparison of the results of laboratory examinations were performed using STATISTICA 13 software (TIBCO Software Inc.). Nonparametric tests were used for comparisons – the Mann-Whitney U test was used for two independent groups, while Spearman’s R was used to assess correlations between two variables. The p-value of <0.05 was considered statistically significant.

## Results

### Clinical characteristics of the patients

The study group consisted of 11 women with excessive GWG and the control group consisted of 10 women with proper GWG.

The main characteristics of the studied group are presented in **Table 1**. The groups did not differ in terms of basic parameters such as the age, gestational age and prepregnancy BMI but, as intended, they significantly differed in GWG.

**Table 1:** Participant characteristics.

Characteristics	Control group (n=10) *Median (IQR)*	Study group (n=11) *Median (IQR)*	p-value
Age [years]	29 (25; 36)	32 (26; 34)	0.83
Gestational age [weeks]	37.29 (35.14; 38.71)	36.57 (34.00; 38.14)	0.50
Prepregnancy BMI [kg/m^2^]	21.36 (19.84; 22.77)	21.60 (20.72; 22.10)	0.92
Gestational weight gain [kg]	11.5 (11.0; 12.0)	18.0 (16.5; 22.0)	<0.01

BMI, Body Mass Index; IQR, interquartile range.

### Bacterial composition overview

**Figure 1:**
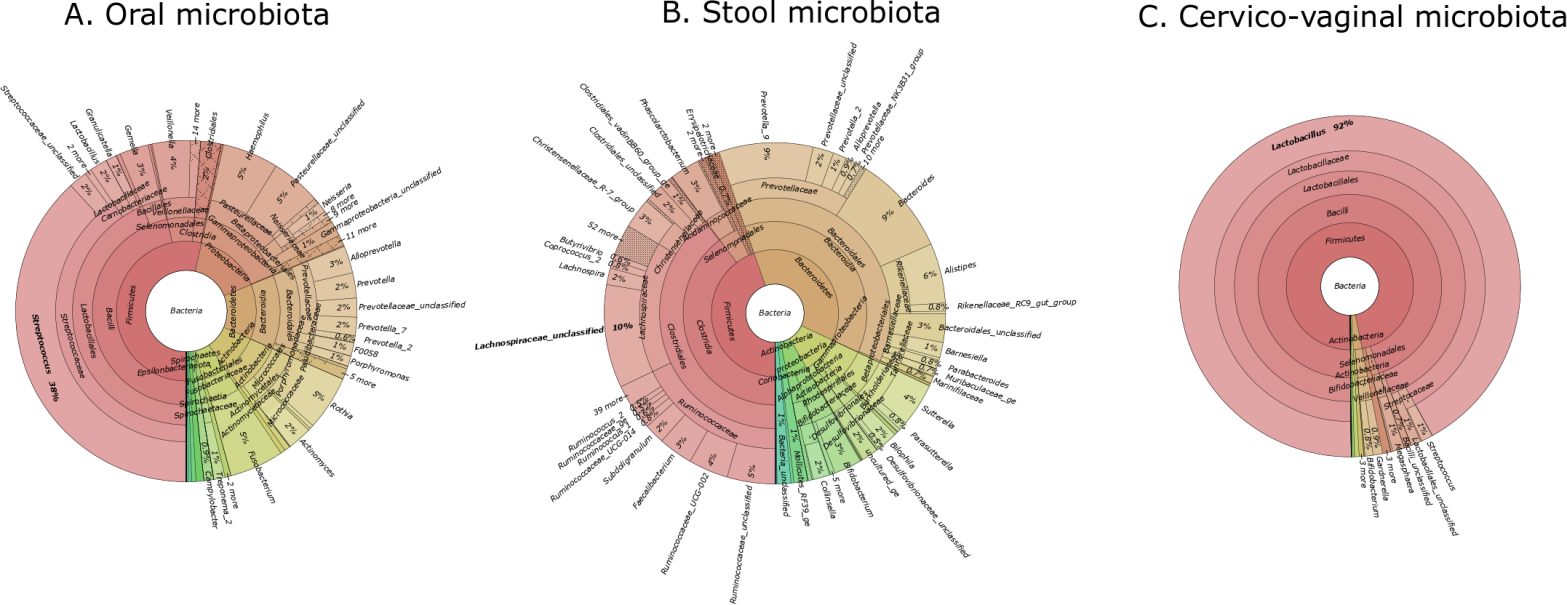
Krona plots showing the taxonomic composition of the microbiota in three body sites. **(A)** oral microbiota, **(B)** stool microbiota, **(C)** cervico-vaginal microbiota. Each chart displays the relative abundance of bacterial taxa at various taxonomic levels. The percentages indicate the proportion of each taxon within the total microbial community for the respective site.

The quantity and quality of DNA isolated from cervical and oral swabs as well as stool samples enabled the construction of 33 and 30 libraries from the study and control group, respectively. The oral, vaginal and fecal microbiota composition of the groups was characterized using 16S rRNA gene sequencing. Of 884 taxa (200 of which were found in over 0.01% of reads), 712/258/435 taxa were present in oral/vain/stool groups, and 177 and 130 were detected only in patients and controls, respectively (**Figure 1**). In total, we detected an average of 151 taxa per sample and 165k reads.

### Cervico-vaginal, oral and stool microbiota alpha and beta diversity metrics

At the genus level, the microbial richness, alpha and beta diversity were estimated using the Chao, Shannon and PCoA indices, respectively.

**Figure 2:**
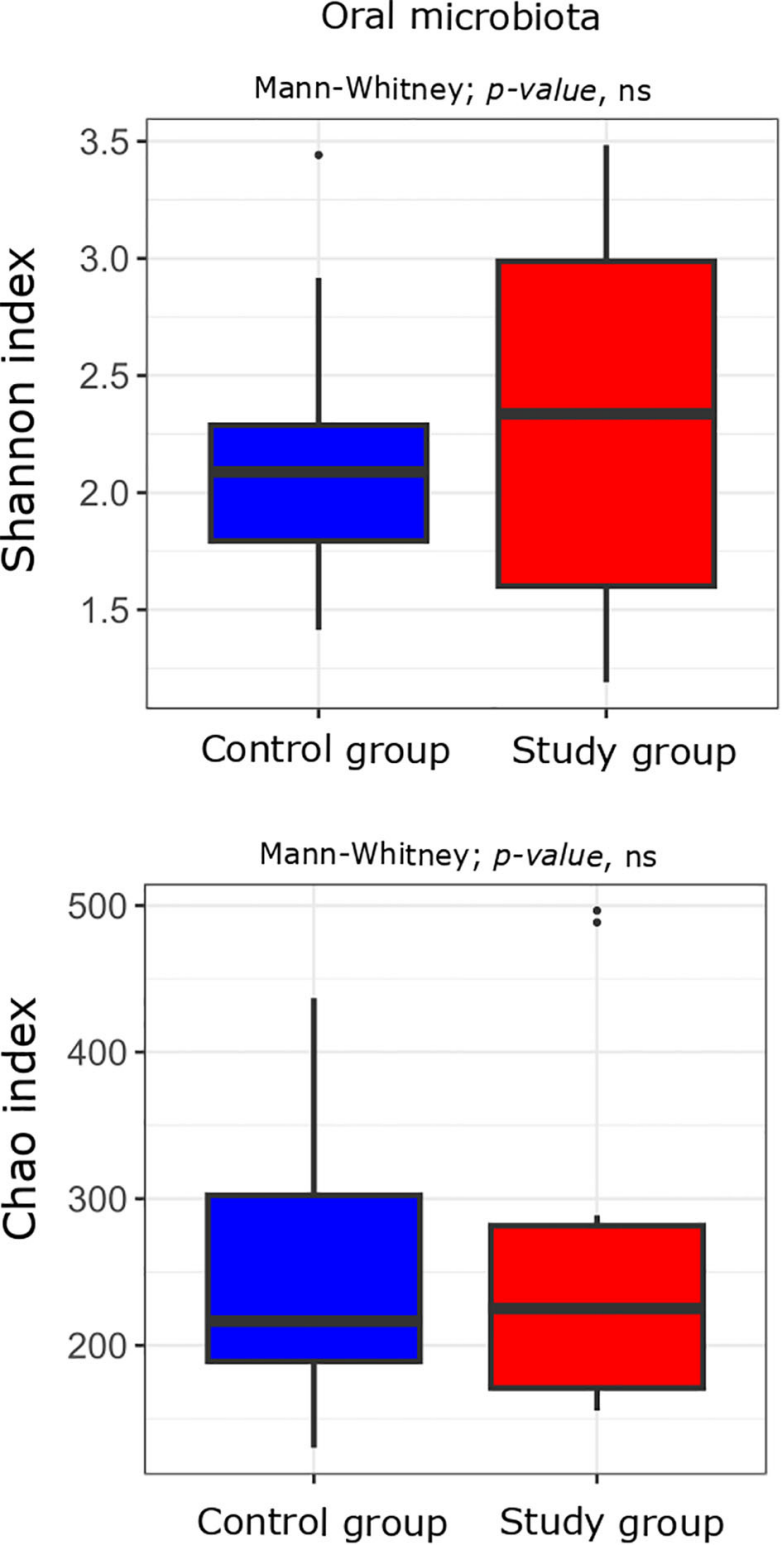
Alpha diversity measured with the Shannon and Chao indices in the oral microbiota of the study (n = 11) and control (n = 10) groups. Comparisons were performed using the Mann-Whitney test; p-values are indicated on the plots. “ns” denotes non-significant differences (p-value > 0.05).

**Figure 3:**
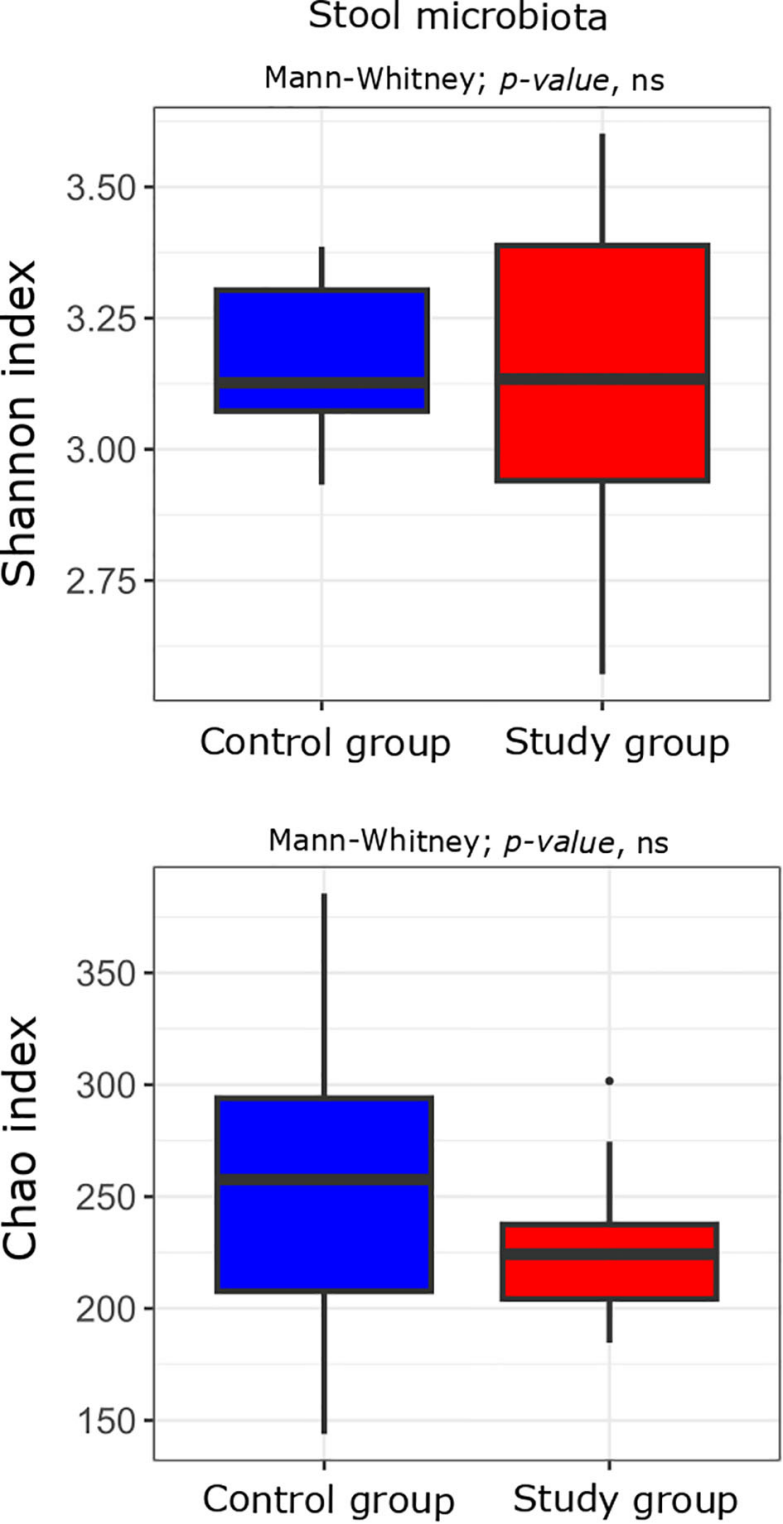
Alpha diversity measured with the Shannon and Chao indices in the stool microbiota of the study (n = 11) and control (n = 10) groups. Comparisons were performed using the Mann–Whitney U test; p-values are indicated on the plots. “ns” denotes non-significant differences (p-value > 0.05).

First, we compared the bacterial alpha diversity of the study group and control patients. Alpha diversity analysis showed no statistically significant differences in the oral and stool microbiota between the study group compared to the controls (**Figures 2**, **3**).

**Figure 4:**
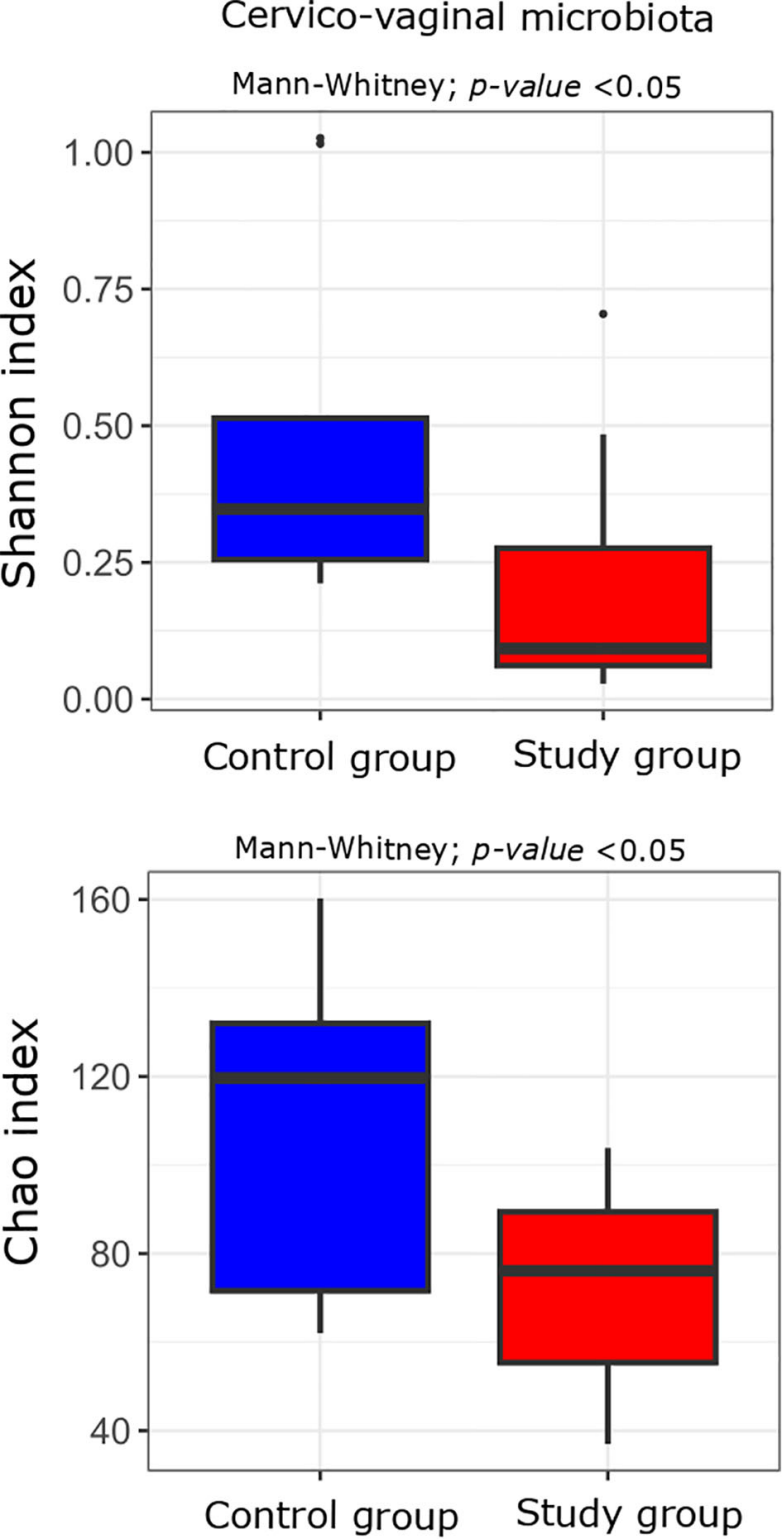
Alpha diversity measured with the Shannon and Chao indices in the cervico-vaginal microbiota of the study (n = 11) and control (n = 10) groups. Comparisons were performed using the Mann-Whitney U test; unadjusted p-values are indicated on the plots. p-value < 0.05 before correction for multiple testing.

At a significance level of p < 0.05 without applying FDR correction, bacterial 16S rRNA gene sequencing analysis of DNA isolated from cervico-vaginal samples revealed a statistically significant decrease in alpha-diversity, measured by the Shannon index (p 0.04), in the group of women with excessive GWG compared to the control group. A similar trend was observed in species richness, assessed by the Chao index (p 0.02). However, after correction for multiple testing, these results were no longer statistically significant, except for the Chao index, which continued to show a tendency toward a decrease (p adj=0.062) (**Figure 4**).

**Figure 5:**
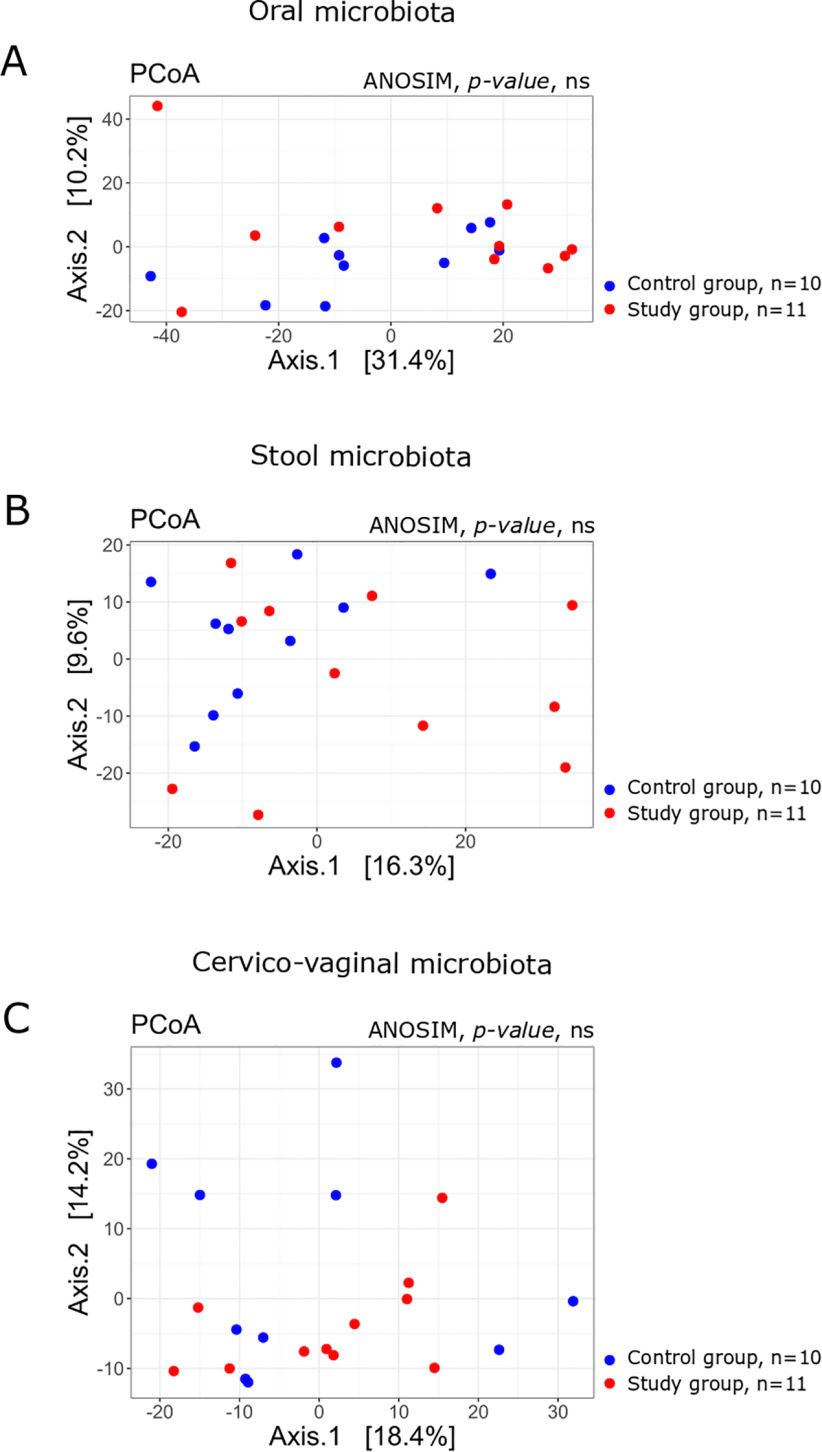
Principal Coordinates Analysis (PCoA) plots based on Bray–Curtis dissimilarity of microbial communities in the **(A)** oral, **(B)** stool, and **(C)** cervico-vaginal samples from the study (n = 11) and control (n = 10) groups. The percentage of variation explained by each axis is indicated. Group separation was assessed using the ANOSIM test; p-values are shown on the plots. Ns, denotes non-significant differences (p > 0.05, unadjusted).

PCoA showed no statistical differences in the beta diversity of oral, cervical and stool microbiota between the study group and the controls (**Figure 5**).

At the genus level, we identified 14 species that significantly differentiated the study from the control group in the oral microbiota, based on p-value analysis. Seven and six of those genera were more (*Faecalibacterium*, *Prevotella*) and less abundant (*Abiotrophia*, *Lactococcus*), respectively, in women with excessive GWG compared to the controls (**Table 2**). However, none of the identified genera remained statistically significant after p-value correction (p adj).

**Table 2:** Bacteria at the genus level differentiating study and control samples in the oral microbiota.

Taxa	baseMean	log2 FC	lfcSE	stat	p-value	p adjusted
*Faecalibacterium*	4.147	2.161258688	0.631954	3.419964	0.002872	0.796028
*Abiotrophia*	154.612	-3.788821563	1.188211	-3.18868	0.004835	0.796028
*Lactococcus*	1179.653	-4.763105529	1.569767	-3.03428	0.006823	0.796028
*Prevotella*	6.115	2.404839069	0.884095	2.720114	0.013586	0.948204
*Sutterella*	4.147	1.883800149	0.694771	2.711397	0.013844	0.948204
*Bacteroides*	10.616	2.127936164	0.808113	2.633217	0.01638	0.948204
*Anaeroglobus*	88.284	-2.11576084	0.827938	-2.55546	0.019334	0.948204
*Alistipes*	9.662	1.575153205	0.672894	2.340865	0.030297	0.948204
*Ruminococcaceae unclassified*	16.416	1.300083572	0.573164	2.268256	0.035162	0.948204
*Mycoplasma*	96.029	-2.709310685	1.208231	-2.24238	0.037063	0.948204
*Aerococcaceae unclassified*	9.754	-1.180835233	0.536646	-2.2004	0.040349	0.948204
*Coprococcus*	4.147	1.464848395	0.673356	2.175444	0.042426	0.948204
*Parasutterella*	4.147	1.294110382	0.605964	2.135624	0.045944	0.948204
*Anaerolineae SBR1031 A4b*	8.316	-0.950791974	0.452403	-2.10165	0.049154	0.948204

baseMean, the average of the normalized count values, divided by size factors, taken over all samples; log2 FC log2 fold change between the groups; lfcSE, standard error of the log2 FC estimate; stat, the value of the test statistic; p-value, p-value of the test; p adjusted, Benjamini–Hochberg-adjusted p-value. Bolded – genus overrepresented in the study group.

We identified 29 genera in the stool samples, with nine and ten being over- and underrepresented, respectively, in intergroup comparisons (**Table 3**). The cervico-vaginal microbiota was characterized by 12 species that differentiated the study group samples from the controls (**Table 4**). Among those, four genera (*Ralstonia, Pandoraea, Kocuria*, and *Rhodobacteraceae unclassified*) were more prevalent in the study group. As in the case of the oral microbiota, none of the identified genera in the stool and cervico-vaginal microbiota was found to be statistically significant after p-value correction (p adj).

**Table 3:** Bacteria at the genus level differentiating study and control samples in the stool microbiota.

Taxa	baseMean	log2 FC	lfcSE	stat	p-value	p adjusted
*Actinobacteria unclassified*	869.282	-2.54587	0.697376	-3.65065	0.001701	0.176438
*Bifidobacteriaceae unclassified*	7977.806	-2.93591	0.809988	-3.62463	0.001804	0.176438
*Bifidobacterium*	54578.49	-2.95627	0.831252	-3.5564	0.002107	0.176438
*Actinobacteria unclassified*	336.2595	-2.45116	0.696727	-3.51811	0.002299	0.176438
*Gammaproteobacteria unclassified*	294.0296	1.565092	0.479511	3.263932	0.004084	0.250744
*Turicibacter*	392.3769	-3.06813	1.013514	-3.02722	0.006931	0.303254
*Mailhella*	9.172253	1.624866	0.545799	2.97704	0.007745	0.303254
*Prevotellaceae unclassified*	478.0516	4.361874	1.469657	2.967954	0.007902	0.303254
*uncultured*	165.8167	-2.44682	0.851005	-2.87521	0.009692	0.330601
*Roseburia*	1368.745	2.134893	0.766015	2.787014	0.011751	0.360763
*Ruminococcaceae UCG-009*	174.0846	-1.8306	0.704656	-2.59786	0.017666	0.493036
*Selenomonadales unclassified*	712.3203	-2.44765	0.989041	-2.47477	0.022926	0.493617
*Family XIII unclassified*	1218.565	-1.5267	0.61855	-2.4682	0.023245	0.493617
*Clostridioides*	37.54836	-1.602	0.654714	-2.44687	0.024308	0.493617
*Erysipelotrichaceae uncultured*	14.94617	4.056052	1.673277	2.424016	0.025497	0.493617
*Christensenellaceae R-7 group*	34958.54	-3.18839	1.333824	-2.39041	0.027344	0.493617
*Ruminococcaceae UCG-011*	61.2443	-1.54813	0.681186	-2.2727	0.034845	0.493617
*Candidatus Soleaferrea*	90.73165	-1.31868	0.581056	-2.26946	0.035076	0.493617
*Alistipes*	112125	-0.9985	0.444984	-2.24391	0.036948	0.493617
*Adlercreutzia*	310.474	-1.87986	0.838308	-2.24244	0.037058	0.493617
*Eubacterium*	9.172253	1.011884	0.453255	2.232482	0.037815	0.493617
*Pseudobutyrivibrio*	9.172253	0.95047	0.432045	2.199933	0.040387	0.493617
*Ruminococcaceae NK4A214 group*	1139.588	-2.2545	1.035445	-2.17732	0.042267	0.493617
*Bacteroidales unclassified*	12070.81	1.230156	0.566192	2.172685	0.042662	0.493617
*Acetanaerobacterium*	27.63764	-1.0278	0.473979	-2.16846	0.043025	0.493617
*Ruminococcaceae UCG-004*	417.12	-1.98198	0.914259	-2.16785	0.043077	0.493617
*uncultured*	1585.828	-3.52393	1.63408	-2.15652	0.044066	0.493617
*Prevotellaceae NK3B31 group*	23.94177	4.22967	1.976185	2.14032	0.045516	0.493617
*Campylobacter*	42.95505	-0.93194	0.437902	-2.12821	0.046628	0.493617

baseMean, the average of the normalized count values, divided by size factors, taken over all samples; log2 FC log2 fold change between the groups; lfcSE, standard error of the log2 FC estimate; stat, the value of the test statistic; p-value, p-value of the test; p adjusted, Benjamini–Hochberg-adjusted p-value. Bolded – genus overrepresented in the study group.

**Table 4:** Bacteria at the genus level differentiating study and control samples in the cervico-vaginal microbiota.

Taxa	baseMean	log2 FC	lfcSE	stat	p-value	p adjusted
*Pandoraea*	9.224582	0.837863	0.272384	3.07604	0.006218	0.308582
*Bacteroides*	97.5528	-1.71087	0.56769	-3.01374	0.007141	0.308582
*Prevotella*	42.74273	-1.63842	0.546633	-2.99729	0.007406	0.308582
*Kocuria*	9.224582	0.837863	0.306197	2.736354	0.013117	0.362148
*Ruminococcaceae unclassified*	46.38516	-1.43083	0.539675	-2.65128	0.015757	0.362148
*Ralstonia*	14.08111	1.36766	0.52493	2.605416	0.017383	0.362148
*Gemella*	31.13965	-1.31357	0.526034	-2.49713	0.021872	0.39058
*Bacilli unclassified*	24029.86	-1.49106	0.634851	-2.34867	0.029814	0.452801
*Faecalibacterium*	36.79929	-1.15826	0.50245	-2.30522	0.032602	0.452801
*Rhodobacteraceae unclassified*	10.42222	0.529676	0.236612	2.238583	0.037349	0.454476
*Clostridiales unclassified*	73.42018	-1.60727	0.737284	-2.17999	0.042041	0.454476
*Firmicutes unclassified*	11964.32	-1.54373	0.727455	-2.1221	0.047198	0.454476

baseMean, the average of the normalized count values, divided by size factors, taken over all samples; log2 FC log2 fold change between the groups; lfcSE, standard error of the log2 FC estimate; stat, the value of the test statistic; p-value, p-value of the test; p adjusted, Benjamini–Hochberg-adjusted p-value. Bolded – genus overrepresented in the study group.

### Bacterial stool metabolite analysis

**Figure 6:**
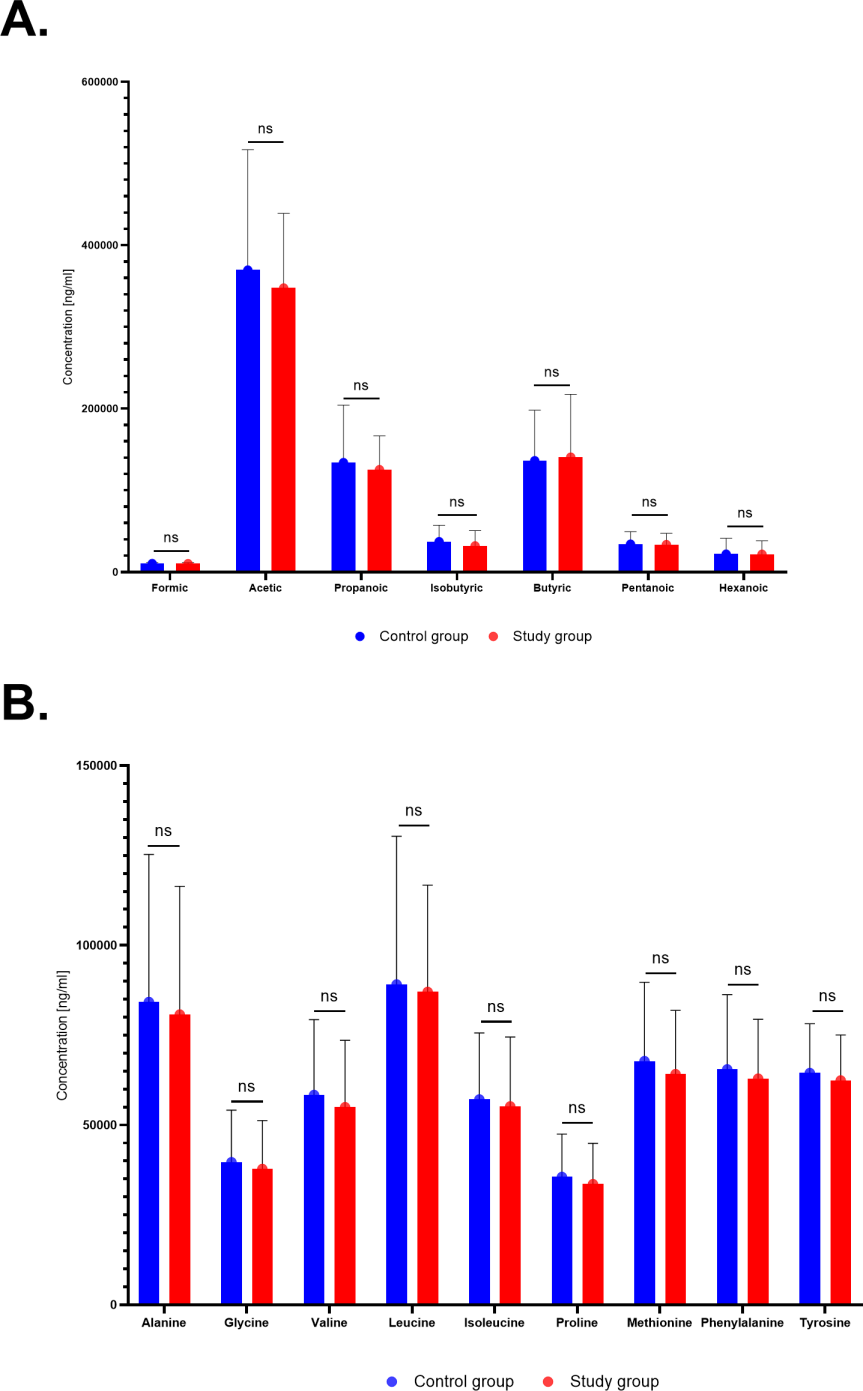
Relative abundance of short-chain fatty acids (SCFAs) **(A)** and amino acids (AAs) **(B)** between the study group and the controls.

In this analysis, we compared the relative concentrations of metabolites per gram of stool mass. Due to the limited sample size, no statistically significant differences were observed between the study and control groups for SCFA and AA analysis (**Figure 6**; **Supplementary Tables 1**, **2**).

## Discussion

### Study findings

The analysis revealed no statistically significant differences in the alpha diversity of the oral and stool microbiota between the study and control groups. However, in cervico-vaginal samples, a decrease in alpha diversity (the Shannon index) was observed in women with excessive GWG compared to the controls, with species richness (the Chao index) showing a similar trend. After multiple testing correction, only the Chao index exhibited a tendency toward borderline significance. At the genus level, several genera were differentially abundant between groups across the oral, stool, and cervico-vaginal microbiota, but none remained statistically significant after p-value adjustment.

The high diversity of bacterial communities was suggested to serve as a proof of a healthy gut ecosystem (Turnbaugh et al., 2009). Data on association between the gut microbiota and maternal obesity are abundant. Exceesive weight was associated with significant microbial changes in the maternal microbiome with increases in Bacteroidetes, Firmicutes, and the Actinobacteria phyla and decreases in Bifidobacteria (Dreisbach et al., 2020). Maternal obesity was linked to a higher abundance of *Staphylococcus aureus, Escherichia coli*, and other *Enterobacteriaceae*, and lower counts of *Bifidobacterium longum* and *Bacteroides fragilis* in previously published studies (Collado et al., 2008; Santacruz et al., 2010).

Conversely, data on associations between the maternal gut microbiome and GWG are scarce and inconclusive. Our study revealed no statistically significant differences in alpha diversity analysis in the stool microbiota of the study group compared to the controls. Similar results were published by Stanislawski et al. They reported no association between postpartum alpha diversity and GWG during pregnancy (Stanislawski et al., 2017). In a study by Kennedy et al., pregnant women with excessive GWG had 11 genera and participants with insufficient GWG had 2 genera that were overall differentially abundant (Kennedy et al., 2023). In this study, excess GWG was linked to a lower abundance of *Prevotella 9* and several SCFA-producing Ruminococcaceae, while insufficient GWG was associated with reduced *Lachnospiraceae NK4A136* group. Among primiparous women, excess GWG was related to decreased *Coprococcus 1*, whereas in multiparous women, excess GWG was associated with increased *Bifidobacterium*, which was generally higher in multiparous than primiparous participants what led to the suggestion that parity may modulate the impact of BMI and GWG on the gut microbiota during human pregnancy (Kennedy et al., 2023).

Some authors reported positive associations between excessive GWG and the abundance of *Clostridium histolyticum*, Bacteroidetes (phylum), *Enterobacter* (genus), *E. coli*, and *Escherichia* spp. and negative associations with the abundance of *Akkermansia muciniphila* and *Bacteroides bacteroide*s (Collado et al., 2008; Collado et al., 2010; Santacruz et al., 2010; Robinson et al., 2017; Smid et al., 2018; Dreisbach et al., 2020). According to Santacruz et al., women with proper GWG had higher numbers of *Bifidobacterium* and *Akkermansia muciniphila* in their stool, while those with excessive GWG had higher fecal numbers of *E. coli* and the abundance of the members in the *Clostridium leptum* subgroup and *Staphylococcus* (Santacruz et al., 2010). A similar relation was reported between excessive GWG and the abundance of organisms in the *Bifidobacteria* genus (Collado et al., 2008; Santacruz et al., 2010; Kennedy et al., 2023). Aatsinki et al. found excessive GWG to be associated with a higher prominence of Bacteroidetes and a lower prominence of Firmicutes compared to women with proper GWG. The authors observed no significant correlations of the abundances of bacterial genera or phyla with GWG. Alpha and beta diversity were also unrelated to GWG. Lower GWG was observed in the subgroup of women with Firmicutes domination and higher GWG was observed in the subgroup with Bacteroidetes domination (15.0 vs. 12.1 kg, respectively, p=0.023). Aatsinki et al. observed the most prominent effect in normal-weight mothers (16.7 vs. 12.5 kg, respectively, p=0.0077) (Aatsinki et al., 2018). On the other hand, Cömert et al. observed an increase in both *Bacteroidetes* and *Firmicutes* phyla when GWG was above the recommended values (Comert et al., 2022). According to Stanislawski, the most important taxa that differentiated GWG study groups (adequate vs. excessive) included members of the genera *Methanobrevibacter*, *Bifidobacterium*, and *Bacteroides*, as well as seven OTUs from the order Clostridiales (Stanislawski et al., 2017).

Bacteroidetes was previously described as associated with the lean phenotype or weight loss (Ley et al., 2006; Turnbaugh et al., 2009). *B. fragilis*, within the Bacteroidetes phylum, was found to correlate with excessive GWG in the third trimester of pregnancy by Collado et al (Collado et al., 2008). Conversely, Santacruz et al., found the number of *B. fragilis* to correlate with normal GWG at 24 weeks of gestation (Santacruz et al., 2010). On the other hand, a negative association was observed between the *Prevotella* genus and excessive GWG (Collado et al., 2008; Kennedy et al., 2023).

Hypotheses explaining the influence of gut microbiota on maternal weight gain include enhanced glucose and fatty acid absorption, increased fasting-induced adipocyte factor release, the activation of catabolic pathways and immune system stimulation (Neuman and Koren, 2017). The gut microbiota may also have an impact on insulin resistance and glucose homeostasis, thereby affecting maternal metabolism (Gomez-Arango et al., 2016).

Bacterial metabolites in the stool were also investigated in relation to GWG. Owing to the small sample size, in this study, the analysis did not reveal any statistically significant differences in SCFAs and AAs between the study and control groups. Kennedy et al. observed different fecal SCFA levels in women with proper and excessive GWG in primiparous women. Fecal acetate was decreased (p=0.04), while propionate was increased (p=0.027) by excessive GWG. Lactate was decreased in primiparas with excessive GWG (p=0.005) (Kennedy et al., 2023). Researchers also found decreased relative abundances of 6 genera of the SCFA-producing family *Ruminococcaceae*, and in *Coprococcus 1*, which had previously been found to be inversely associated with circulating triglyceride levels in nonpregnant individuals (Fu et al., 2015).

Data on the cervico-vaginal microbiota and maternal overweight or obesity and GWG are very limited. In our study, a decreasing trend in alpha diversity measured with the Shannon index and in species richness assessed with the Chao index were observed in women with excessive GWG compared to the control group. Ingram et al. collected vaginal samples at 10–14, 18–24, 26–30, and 34–37 weeks of gestation and at delivery from normal-weight, overweight and obese women. They found vaginal bacterial alpha diversity to be higher in obese participants (p=0.033). The relative abundances of *Peptoniphilus* and *Anaerococcus* were increased in overweight and obese pregnant women (Ingram et al., 2024). Data on the cervico-vaginal microbiota in relation to excessive GWG remain scarce. Most available studies focus on maternal BMI rather than weight gain during pregnancy. In our study, a decreasing trend in alpha diversity measured with the Shannon index and in species richness assessed with the Chao index was observed in women with excessive GWG compared to the control group. Ingram et al. collected vaginal samples at multiple time points during pregnancy and found bacterial alpha diversity to be higher in obese participants, with increased relative abundances of *Peptoniphilus* and *Anaerococcus* (Ingram et al., 2024). The observed trend toward decreased alpha diversity and richness in women with excessive GWG may indicate a distinct microbial pattern associated with gestational weight gain, although further studies are needed to confirm this association.

The oral cavity contains the second most complex microbial population within the human body. The total viable microbial counts in pregnant women are known to be higher compared to non-pregnant women (Jang et al., 2021; Saadaoui et al., 2021). Studies revealed a significant increase in *Streptococcus mutans, Aggregatibacter actinomycetemcomitans*, *Porphyromonas gingivalis* and *Prevotella intermedia* in the oral cavity of pregnant women (Gursoy et al., 2009; Emmatty et al., 2013; Kamate et al., 2017). Numerous authors investigated associations between the oral micriobiome and pregnancy outcome. High levels of periodontal pathogens, especially *P. gingivalis*, were associated with an increased risk for preterm delivery (Costa et al., 2019). An increased number of *P. gingivalis* and *E. corrodens* were observed in women with pre-eclampsia who developed an adverse birth outcome (Contreras et al., 2006). Conversely, relations between the oral micriobiota and GWG or obesity during pregnancy have not been investigated until now. We found significant differences in the oral microbiome between patients with proper and excessive GWG. Some genera were more (*Faecalibacterium*, *Prevotella*) or less abundant (*Abiotrophia*, *Lactococcus*), respectively, in women with excessive GWG.

Although our study did not demonstrate statistically significant differences in microbial diversity or metabolite levels between groups, the observed trends and previously published associations suggest that maternal microbiota may play a role in gestational weight gain and pregnancy outcomes (Sinha et al., 2023). These findings underscore the need for future research into microbiota-targeted interventions, such as dietary modifications, probiotic or prebiotic supplementation, and lifestyle strategies aimed at modulating the maternal microbiome. Screening for specific microbial patterns during pregnancy could potentially help identify women at risk of excessive GWG or related complications, paving the way for personalized maternal care approaches (Geyer et al., 2023).

The strengths of the study include its prospective nature and complex analysis of the microbiota of pregnant women. To our knowledge, a unique analysis of the gut, cervico-vaginal and oral mirobiota in pregnant women has been the first one to be published to date. Exclusion criteria including antibiotic and probiotic use aimed to recruit a homogenous group of subjects. The metagenomic results are characterized by a large diversity both in our study and in previously published reports. Poland is considered an ethnically homogeneous country. Therefore, this factor is unlikely to be a significant source of bias. However, our study is not devoid of limitations. Gut microbiota composition is influenced by the diet. Diet is one of the key factors influencing the composition and function of the gut microbiome. Variations in the intake of fiber, fats, proteins, and fermentable substrates significantly affect microbial diversity and the dominance of specific bacterial taxa. Even short-term dietary changes can result in measurable shifts in the microbiome profile. Regrettably, dietary information was unavailable. Therefore, correlations between dietary intake and the microbiome in women with proper and excessive GWG could not be analyzed. Due to the lack of detailed dietary information in our cohort, the correlation analyses between the microbiome and metabolome should be considered preliminary. These findings require cautious interpretation and validation in larger, well-controlled studies. Future research with comprehensive dietary assessment will be essential to confirm and refine these associations. Our study used a small sample size, so the results may not represent the whole population of pregnant women. The number of participants was constrained by the available funding and therefore may introduce bias. Obtaining statistically significant results in small study groups may be impossible. Despite that, we demonstrated a certain trend in alpha diversity, measured with the Shannon and Chao indices in the cervico-vaginal microbiota. We believe that being a pilot study, it will help to inform and refine future, resource-demanding research efforts. Further prospective studies on larger group samples are subsequently needed to fully understand correlations between the microbiome and GWG in pregnant women. Additionally, due to the limited sample size and the low number of taxa showing statistically significant differences between groups, we did not perform correlation analyses between microbial taxa and SCFA or amino acid levels. We considered such analyses to be underpowered and potentially misleading. This limitation should be addressed in future studies with larger cohorts and integrated microbiome-metabolome datasets.

## Conclusions

Our analysis revealed slight trends in microbiota composition, despite the limitations imposed by the small sample size. In cervico-vaginal samples, bacterial 16S rRNA gene sequencing demonstrated a decrease in alpha diversity, measured with the Shannon index, among women with excessive GWG compared to the control group. While this difference was not statistically significant after correction for multiple testing, the Chao index showed a persistent trend toward reduced species richness in the study group. In stool samples, we identified 29 genera with differential representation between the groups, including nine overrepresented and ten underrepresented genera. Additionally, the cervico-vaginal microbiota analysis identified 12 species distinguishing the study group from the controls, with four genera (Ralstonia, Pandoraea, Kocuria, and Rhodobacteraceae unclassified) being more prevalent in the study group. Nevertheless, in both sites none difference was found to be statistically significant after p-value correction. These findings suggest potential differences in microbial diversity and composition associated with excessive GWG, supporting further investigation into their role in maternal health.

## Data Availability

The original contributions presented in the study are included in the article/**Supplementary Material**. Further inquiries can be directed to the corresponding author.
