# Supplementary material for: Analysis of the microbiota of pregnant women in relation to weight gain during pregnancy – a pilot study
Source: Front Cell Infect Microbiol. 2025 Aug 29;15:1655581. doi: 10.3389/fcimb.2025.1655581 (PMC12426079; doi:10.3389/fcimb.2025.1655581)
Supplement: Supplementary file 1 [file Table1.docx]

Supplementary Table 1. Results of aminoacids determinations in the tested stool samples.

| Parameter | Control group (n=10)  Median (IQR) | Study group (n=11)  Median (IQR) | p-value |
| --- | --- | --- | --- |
| Alanine [ng/ml] | 71526.72  (56465.00; 104057.06) | 90978.14  (44780.72; 114064.30) | 1.00 |
| Glycine [ng/ml] | 35319.44  (32781.73; 44054.62) | 40353.61  (23335.42; 49429.44) | 0.85 |
| Valine [ng/ml] | 54900.40  (42998.68; 64469.84) | 59177.95  (35195.54; 67588.37) | 0.97 |
| Leucine [ng/ml] | 84917.63  (59658.99; 95361.05) | 94341.04  (59758.24; 110832.56) | 0.62 |
| Izoleucine [ng/ml] | 55273.42  (43635.73; 62174.39) | 57356.72  (35086.22; 68572.87) | 0.97 |
| Proline [ng/ml] | 34551.15  (30454.35; 39946.89) | 31284.88  (23779.73; 44619.58) | 0.85 |
| Methionine [ng/ml] | 61077.27  (49658.20; 79311.61) | 68268.03  (44434.52; 75942.90) | 0.85 |
| Phenylalanine [ng/ml] | 64433.16  (50058.10; 76478.10) | 69728.80  (44383.69; 79020.38) | 0.79 |
| Tyrosine [ng/ml] | 64424.74  (53831.15; 70515.97) | 63047.80  (48861.82; 75277.91) | 0.79 |

IQR – interquartile range (przedział międzykwartylowy)

Supplementary Table 2. Results of organic acids determinations in the tested stool samples.

| Parameter | Control group (n=10)  Median (IQR) | Study group (n=11)  Median (IQR) | p-value |
| --- | --- | --- | --- |
| Formic acid [ng/ml] | 10215.35  (9640.92; 10870.83) | 10463.98  (9054.53; 11656.39) | 0.97 |
| Acetic acid [ng/ml] | 344097.71  (244571.04; 434487.24) | 355911.00  (291060.37; 431109.40) | 0.91 |
| Propionic acid [ng/ml] | 120423.05  (75213.15; 189795.04) | 116142.70  (98822.34; 147807.16) | 0.97 |
| Isobutyric acid [ng/ml] | 28021.65  (26085.26; 43763.88) | 30474.52  (19030.51; 52456.60) | 0.97 |
| Butyric acid [ng/ml] | 114060.38  (95833.77; 185047.09) | 111148.01  (70457.95; 194727.27) | 0.52 |
| Valeric acid [ng/ml] | 33007.57  (23089.98; 37960.47) | 35055.91  (20950.85; 41400.43) | 0.97 |
| Caproic acid [ng/ml] | 18598.87  (11909.32; 30333.50) | 19518.30  (48690.25; 30572.51) | 0.68 |

IQR – interquartile range
